# Characterization of Key Odorants in Jimo Huangjiu Using a Characteristic Aroma-Directed Screening Strategy

**DOI:** 10.3390/foods15061111

**Published:** 2026-03-23

**Authors:** Hongtao Yu, Siman Zheng, Liuxi Chen, Juan Wang, Hongqin Liu, Jinglin Zhang, Mingquan Huang, Jihong Wu, Dongrui Zhao, Jinchen Li

**Affiliations:** 1 Key Laboratory of Brewing Molecular Engineering of China Light Industry, Beijing Technology & Business University (BTBU), Beijing 100048, China; yht31@aliyun.com (H.Y.); siman9912@163.com (S.Z.); clx286090@163.com (L.C.); zhjinglin0130@163.com (J.Z.); hmqsir@163.com (M.H.); wujihong12@126.com (J.W.); zdr@btbu.edu.cn (D.Z.); lijinchen@btbu.edu.cn (J.L.); 2 Niulanshan Distillery, Beijing Shunxin Agriculture Co., Ltd., Beijing 101301, China; 3 Institute of Agri-Food Processing and Nutrition, Beijing Academy of Agriculture and Forestry Sciences, Beijing 100097, China

**Keywords:** Jimo Huangjiu, key odorants, pyrazines, phenolic compounds, synergistic interactions

## Abstract

Jimo Huangjiu (JMHJ), a Chinese geographical indication product from Shandong Province, is characterized by distinctive burnt-like and smoky aromas. However, the specific odorants responsible for these sensory attributes remain uncharacterized. In this study, the flavor characteristics of Jimo Huangjiu are characterized through static and dynamic sensory evaluation during the drinking process. This study identified the essential odorants of JMHJ through integrated sensomics analysis. Results revealed pyrazines and phenolic compounds as the characteristic aroma markers responsible for the unique smoky and burnt-like aroma of JMHJ. Ethyl 2-methylpropionate, 4-methylphenol, 4-ethyl-2-methoxyphenol, *β*-phenylethyl alcohol, 2-ethyl-6-methylpyrazine, 2-ethyl-3-methylpyrazine, 2-methylpyrazine, 2-methoxyphenol, 2-methylphenol, 2,3-dimethylpyrazine, and 2-hydroxy-3-methyl-2-cyclopentenone were confirmed as key odorants in JMHJ. Furthermore, the synergistic interactions between nonanoic acid and phenolic compounds were found to contribute to a Qu-like aroma, representing a novel mechanism for this characteristic sensory attribute in Huangjiu.

## 1. Introduction

As a traditional Chinese alcohol beverage, Huangjiu is widely recognized as one of the earliest fermented drinks in human history, together with wine and beer. Its alcohol content generally falls within the range of 8% to 18% (*v*/*v*) [1,2]. Rice, corn, foxtail millet, broomcorn millet, and oats are commonly used as raw materials in Huangjiu production. The basic production processes include steaming, boiling, or soaking these materials, followed by cooling, adding Qu, primary fermentation, chief fermentation, Kaipa (stirring), post-fermentation, press filtration, boiling liquor (sterilization), storage, blending, and packing [2,3]. Simultaneous saccharification and fermentation constitutes a basic process in Huangjiu brewing, during which starch in the materials are continuously hydrolyzed into sugars by enzyme-rich Qu and then converted into ethanol through fermentation, thereby contributing to the broad alcohol range observed among different Huangjiu styles [1]. Wheat Qu, Xiaoqu (Jiuyao), and Hongqu are the principal fermentation starters used in Huangjiu brewing. These starters differ in substrate composition. Wheat Qu is produced mainly from wheat, whereas Xiaoqu (Jiuyao) and Hongqu are commonly prepared from rice and rice/bran mixtures [4]. These starters exhibit distinct dominant microbiota and enzymatic characteristics, which shape different metabolic pathways and metabolite profiles during fermentation. Wheat Qu is commonly associated with Bacillus species and filamentous fungi such as Aspergillus- or Rhizopus-like taxa, whereas Hongqu is dominated by Monascus-related molds along with coexisting bacteria (e.g., lactic acid bacteria) [4]. Xiaoqu (Jiuyao) often contains yeasts (e.g., *Saccharomycopsis*) and lactic acid/acetic acid bacteria, along with saccharifying molds [5]. Based on the production processes and product characteristics of Huangjiu, it can be classified by its total sugar content, the type of Qu used, raw materials, geographical origin, and processing methods. Huangjiu is categorized into dry (≤15.0 g/L), semi-dry (15.1–40.0 g/L), semi-sweet (40.1–100.0 g/L), and sweet (>100.0 g/L) types according to their total sugar content [6]. Similarly, Huangjiu can be classified by raw materials, including corn, foxtail millet, broomcorn millet, oats, buckwheat, and glutinous rice, as well as its geographical region of origin: Shaoxing, Fangxian, Daixian, and Jimo Huangjiu [7]. Generally, southern Chinese Huangjiu is predominantly made from rice, whereas northern Huangjiu is often produced from various blends of local cereals, including quinoa, broomcorn millet, corn, or oats.

The aroma profile of Huangjiu directly influences consumer purchasing decisions. The overall aroma of Huangjiu comprises sweetness, sauce, rice, and alcoholic aromas. However, each type of Huangjiu also offers distinct aromas. Among all types of Huangjiu, JMHJ from Shandong Province stands out due to its prominent smoky and burnt-like aromas, distinguishing it from other varieties. This characteristic aroma is instantly recognizable and memorable to consumers and closely related to the unique production process of JMHJ. Unlike other Huangjius, JMHJ is brewed from broomcorn millet (*Panicum miliaceum* L.) with wheat Qu. During the cooking stage of the broomcorn millet, the JMHJ production process requires the millet to be heated until it becomes charred but not burnt, resulting in a characteristic brownish-red color. In contrast, most other types of Huangjiu only cook the rice until it is fully gelatinized at this stage, without any charring involved. This difference is particularly evident in various southern types of Huangjiu, such as Shaoxing Huangjiu, which predominantly uses the rice steaming method [8].

Recent studies have systematically investigated the key aroma profiles of different types of Huangjiu [1,9,10,11,12,13,14,15,16,17,18]. To investigate aroma-active constituents, researchers have applied several extraction strategies, such as solid-phase extraction (SPE) [1], solid-phase microextraction (SPME) [9], solvent-assisted flavor evaporation (SAFE) [10], and simultaneous distillation/extraction (SDE) [11]. These methods are typically coupled with instrumental and sensory analytical techniques, including gas chromatography–olfactometry (GC–O), comprehensive two-dimensional gas chromatography–mass spectrometry (GC×GC–MS), and gas chromatography–mass spectrometry (GC–MS) [12], together with aroma reconstitution and omission/addition tests. Using these approaches, a number of compounds are recognized as the key contributors to the characteristic aroma of Shaoxing Huangjiu and coarse-grain Huangjiu produced in Daixian, Hebei, and other regions. Among the principal odorants identified are vanillin, sotolon, 2-phenylethanol, 2-acetyl-1-pyrroline, 1,1-diethoxyethane, 3-(methylsulfanyl)propanal, 3-methylbutanal, and 3-methylbutanoic acid [9,12,13,14,15,16,17,18]. In contrast, studies on JMHJ remain scarce, and its key aroma-active compounds, particularly those responsible for the distinctive smoky and burnt-like notes, have not yet been characterized. Among the limited literature, Yan et al. [19] examined volatile compounds and their changes during aging in JMHJ employing HS-SPME and GC-MS. Jiang et al. [8] employed a combination of HS-SPME-ARROW and SAFE pretreatment techniques coupled with GC×GC-TOFMS to investigate the effects of the *Zhumi* (boiling millet) step on the volatile composition of materials used for JMHJ. They identified 86 and 403 volatile compounds before and after boiling, respectively. However, the study only provided a qualitative profile of volatile compounds in the materials of JMHJ. It remains unclear which of these compounds are metabolized or transformed during Huangjiu fermentation, which are retained in the final product, what their concentrations are in Huangjiu, and how much they contribute to the overall aroma. Importantly, a comprehensive volatile list alone is insufficient to pinpoint the odorants responsible for the distinctive smoky and burnt-like notes in Huangjiu, because aroma impact depends on both concentration and odor threshold and requires aroma-focused validation (e.g., GC–O and sensomics-based approaches). Therefore, elucidating the essential odorants contributing to the characteristic smoky and burnt-like aroma of JMHJ remains necessary.

In addition, previous studies on Huangjiu aroma have relied on static sensory evaluations [9,13,14,15,16,17]. However, Huangjiu is consumed as a beverage and typically exhibits pronounced changes in perceived aroma and taste from the first sip through swallowing and aftertaste, which may not be fully captured by static sensory profiling. Temporal Dominance of Sensations (TDSs) is a dynamic sensory approach that tracks the dominant sensation over time and has been successfully applied to assess temporal perception in various food and beverage matrices [20,21,22]. TDS offers a promising tool to complement static sensory profiling and better link dynamic perception during drinking with the identification of key odorants in JMHJ.

Therefore, this paper investigated the key aroma-active compounds responsible for the distinctive characteristics of JMHJ through the following approaches: (1) profiling the orthonasal and retronasal aromas of JMHJ; (2) extracting and screening odor-active substances using SPE coupled with gas chromatography–olfactometry–mass spectrometry (GC–O–MS) and fast-track aroma extract dilution analysis (AEDA); (3) determining the concentrations of the identified compounds and determining their odor activity values (OAVs); (4) validating the roles of critical odorants through aroma recombination; and (5) confirming the key contributors to the smoky and burnt-like aroma attributes of JMHJ by means of addition experiments.

## 2. Materials and Methods

### 2.1. Materials and Chemicals

Three JMHJs were selected to represent the major commercial styles of Jimo Huangjiu, which have distinct levels of sweetness (dry, semi-sweet, and sweet) based on total sugar content. Jimo dry-type Huangjiu (JM-G, sugar content ≤ 15.0 g/L), Jimo semi-sweet-type Huangjiu (JM-BT, sugar content < 40.1~100.0 g/L), and Jimo sweet-type Huangjiu (JM-T, sugar content > 100.0 g/L) were purchased from Shandong Jimo Brewing Co., Ltd., Qingdao, China. The alcohol contents of the three Huangjius were as follows: JM-G, 11.5% (*v*/*v*); JM-BT, 11.5% (*v*/*v*); and JM-T, 11.5% (*v*/*v*).

JMHJ is traditionally produced from millet-based materials through a sequence of operations including soaking/steaming of grains, the zhumi (boiling millet) step, cooling, addition of wheat Qu, primary fermentation, main fermentation with periodic stirring (Kaipa), post-fermentation, pressing, filtration, boiling liquor (sterilization), storage, blending, and packing [8]. During the zhumi step, thermal responses such as Maillard and Strecker reactions between reducing amino acids and sugars may generate or increase nitrogen-containing volatiles and other aroma-active compounds. During subsequent fermentation and vinification, Qu-derived enzymes and microbial metabolism can further generate, transform, or degrade aroma compounds through pathways including amino-acid catabolism, esterification, oxidation/reduction, and aging-related transformations [23,24,25,26,27,28]. As this study focuses on aroma characterization and key-odorant identification in JMHJ rather than process tracking, these pathways should be considered plausible contributors, as quantitative attribution has not been confirmed.

All standard substances (purities above 95%) are summarized in Appendix A. The C6–C26 n-alkane mixture was procured from Sigma-Aldrich, Shanghai, China. HPLC-grade dichloromethane (99.99%) was obtained from Thermo Fisher Scientific, Beijing, China. Absolute ethanol (99.9%), together with anhydrous sodium sulfate (99.8%) and sodium chloride (99.8%), as well as hydrochloric acid at 36.0–38.0%, were all supplied by Sinopharm Chemical Reagent Co., Ltd., Beijing, China.

### 2.2. Sensory Evaluation

#### 2.2.1. Quantitative Descriptive Analysis (QDA)

QDA was employed to evaluate the orthonasal aroma profiles of different Huangjiu samples [29]. The sensory panel consisted of 30 individuals, with an equal distribution of males and females (15 each), aged 22–30 years. All panelists had prior training or experience in sensory evaluation and quantitative descriptive analysis and were enlisted from the Key Laboratory of Brewing Molecular Engineering, Light Industry, China. The panelists underwent a three-week training program (30 min per day), during which they learned to recognize and describe 54 standard aroma solutions and to distinguish differences among aroma attributes. Based on their individual sensitivity to aroma and the accuracy of their description, 14 panelists (7 females and 7 males) were ultimately nominated for the formal assessment.

At the beginning of the evaluation, 15.0 mL samples of each type of Huangjiu were added into a 50.0 mL glass bottle labeled with a randomly assigned three-digit code. All samples were served to the assessors according to a random presentation sequence. Each panelist was instructed to use 6 to 9 descriptive terms to record the sensory characteristics of each sample. Sensory descriptors used in QDA refer exclusively to orthonasal aroma perception (headspace aroma of the whole Huangjiu sample) and do not include taste or mouthfeel attributes.

Subsequently, descriptors that occurred with higher frequencies were screened and discussed by the sensory panel until a consensus was reached regarding the final set of aroma descriptors. Consequently, burnt-like, smoky, acidic, sweet, Chen (aged aroma), alcoholic, fermented, and woody characteristics were identified as the most frequently perceived aroma attributes in JMHJ. The reference standards for these aroma attributes are shown in Appendix A (Table A1), adapted from the literature with appropriate modifications [9].

Finally, the Huangjiu samples were re-evaluated by trained panelists. Each panelist rated the intensity of eight aroma attributes using a nine-point scale based on the reference standards outlined in Table A1. Scores of 1–3, 4–6, and 7–9 represented weak, moderate, and strong intensities, respectively. All sensory evaluations were conducted in a controlled environment with a relative humidity of 45–50% at 21 ± 1 °C. Each panelist performed triplicate evaluations, with variation among replicates required to be within 20%.

#### 2.2.2. Dynamic Aroma Perception Evaluation

The aroma perception of JMHJ was evaluated using the Temporal Dominance of Sensations (TDS) method to analyze the dynamic changes in aroma characteristics during tasting [20,29]. In this test, panelists were instructed to only identify the prevailing aroma attributes perceived at each moment throughout the tasting process, without assessing their intensity [20,30,31]. The same trained panelists described in Section 2.2.1 participated in this experiment. In the TDS evaluation, data acquisition was focused on the post-swallowing phase to capture retronasal perception. The moment of swallowing was defined as t = 0 s, and panelists began attribute selection immediately after swallowing; therefore, 2 s represents the time point right after swallowing. The TDS attribute list included both retronasal aroma, taste, and mouthfeel descriptors.

Specifically, initial preliminary measurements were conducted to determine the average drinking duration for a 15 mL sample of JMHJ. The time from sample intake to swallowing was approximately 2 s, and the subsequent retronasal perception of aroma after swallowing was recorded. The total average duration of aroma perception was found to be 120 s. Based on these results and discussions within the sensory panel, seven time points (2, 20, 40, 60, 80, 100, and 120 s) were selected to evaluate both aroma and taste perception throughout the tasting process.

Due to the brief tasting duration and potential interaction between samples and saliva affecting aroma release, the retronasal aroma profile after swallowing differed significantly from the orthonasal aroma. Therefore, the Check-All-That-Apply (CATA) method was further employed to determine the specific aroma and taste attributes perceived during tasting. Terms describing the aroma were selected based on the 20 descriptors identified in the QDA. Taste attributes encompassed saltiness, bitterness, sweetness, sourness, astringency, and umami. The final selection of aroma and taste attributes was established through statistical screening and discussions among panelists.

The TDS curves were generated by plotting the dominance rates (%) of each sensory attribute at different time points. All panelists were instructed to refrain from consuming food for at least one hour before to the evaluation. Before the formal test, they underwent two weeks of TDS training and pre-testing to familiarize themselves with the procedure. Each experiment was conducted in triplicate.

The principle of TDS analysis is based on the probabilistic identification of dominant sensory attributes at specific time points. Thus, statistical screening is required to determine which dominant results are significant. For clearer interpretation of the TDS visualization, two benchmark lines are introduced into the graph. The first corresponds to the chance level and reflects the dominance proportion that may arise randomly for a given attribute. It is expressed as P_0_ = 1/*p*, with *p* referring to the number of evaluated attributes. Another benchmark is the significance level, denoted as P_S_, defining the minimum dominance proportion required for an attribute to be considered significantly above chance. Its value is estimated from the confidence interval for a binomial proportion under the normal approximation [Equation (1)] [20].

(1) $$P_{s}=P_{0}+1.645\times \sqrt{\frac{P_{0}(1-P_{0})}{n}}$$

Here, the coefficient 1.645 corresponds to the one-sided 95th percentile of the standard normal distribution. Specifically, for Z~N (0,1), the value z is such that Pr(Z ≤ z) = 0.95, resulting in z ≈ 1.645. In the TDS framework, this constant is used to define a one-sided significance threshold above the chance level P_0_ = 1/p (with p sensory attributes). The inferential question is directional: it asks whether the observed dominance rate at a given time is greater than what would be expected under random attribute selection—for instance, a right-tailed test at α = 0.05 naturally yields the critical value z1-α = z0.95 = 1.645 (whereas a two-sided 95% criterion would equal 1.96). It should be noted that this threshold is best interpreted as a visual, pointwise reference line rather than as strict per-time point inference, because TDS observations typically involve repeated measures and temporal dependence (adjacent time points are correlated). Likewise, simultaneous evaluations across multiple attributes and time points introduce a multiple-comparisons concern. Accordingly, a more robust interpretation emphasizes contiguous time intervals during which dominance curves exceed the threshold, rather than isolated exceedances at single time points [20].

### 2.3. Isolation of the Volatiles

The aroma compounds in JMHJ were isolated by solid-phase extraction (SPE), following a previously reported procedure with slight modifications [1]. Briefly, a 20 mL JMHJ sample was mixed with 20.0 µL of 2-methyl-3-heptanone (1000 mg/L, internal standard) and 3.0 g of NaCl. This mixture was then loaded onto an SPE column that had been preconditioned sequentially with 6.0 mL of dichloromethane, methanol, and ultrapure water. The loading flow rate was maintained at less than 2 mL/min. After sample application, the SPE column was washed with 6.0 mL of ultrapure water and allowed to dry. Elution of the target analytes was subsequently conducted using 6.0 mL of dichloromethane. The collected eluent was dehydrated with anhydrous sodium sulfate, then reduced to a final volume of 0.50 mL under a gentle stream of nitrogen (99.999%, 10 mL/min), and kept at −40 °C until further analyses.

### 2.4. Gas Chromatography–Olfactometry–Mass Spectrometry (GC-O-MS)

The concentrated extracts were subsequently subjected to GC–MS analysis using a 7890B GC System coupled to a 5977A MSD and an olfactory detection port (ODP 3, Gerstel, Germany). For analysis, 1.0 μL of each concentrated extract was introduced onto a DB-WAX capillary column (30 m × 250 μm × 0.25 μm; Agilent Technologies, Santa Clara, CA, USA). Helium with a purity of 99.999% served as the carrier gas at a steady flow rate of 2.0 mL/min. The injector temperature was maintained at 250 °C. The oven program started at 40 °C, followed by heating to 50 °C at 10 °C/min with a holding time of 10 min, followed by 80 °C at 3 °C/min with another 10 min hold, and eventually to 230 °C at the pace of 5 °C/min, where it was maintained for 2 min. After chromatographic separation, the column effluent was equally divided between the mass spectrometer and the olfactory detection port at a split ratio of 1:1 (*v*/*v*). The transfer line temperature was set to 250 °C, whereas both the ion source of the ODP and the mass spectrometer were maintained at 230 °C. Mass spectra were recorded under electron impact ionization at 70 eV, and full-scan data were collected over an *m*/*z* range of 30–350 [12].

In GC-O, odor descriptors were recorded as compound-level orthonasal perceptions at the GC effluent during sniffing; they were used to describe the odor quality of individual odor-active peaks. GC–O assessment was carried out by a qualified sensory panel consisting of three people (two females and one male). To improve their ability to recognize volatile constituents, the panelists underwent training with no fewer than 40 odor-active compounds presented at concentrations fivefold greater than the odor threshold values in air (Table 1). Repeated evaluations were carried out until the results showed satisfactory reproducibility. Aroma compounds were initially characterized through comparison of their retention indices (RIs) and mass spectra with entries in the NIST (2022) database and the Baijiu Flavor Compounds database created by our research team. Further confirmation was achieved by matching the spectral features and odor properties of the detected compounds against the respective authentic standards [12]. The RIs were calculated utilizing a customized Kovats method [32]. Odor descriptors for each odor-active peak were assigned by panel consensus following training with authentic reference standards [12].

### 2.5. Odor-Specific Magnitude Estimation (Osme) and Fast-Track Aroma Extract Dilution Analysis (Fast-Track AEDA)

Employing the GC–O–MS approach described above, an Osme analysis was conducted by a sensory panel composed of three evaluators, including two women and one man. For data collection, only odor zones perceived by at least two of the three panelists were considered valid. The measurements were repeated until good reproducibility was obtained. During each run, aroma intensity (AI) was evaluated on a scale from 0 to 5, where 0 indicated the absence of odor, 3 denoted a moderate perception, and 5 indicated extremely high intensity. All samples were sniffed in triplicate by each evaluator [12,33].

Based on previous methodologies, a simplified approach termed fast-track AEDA was developed and used in the study. Unlike the conventional AEDA, which employs a series of 2-fold dilutions for olfactometry, the fast-track method utilizes 10-fold dilutions in only two steps (10× and 100×) to rapidly screen for the most potent aroma-active compounds. The concentrated extracts were diluted ten-fold and hundred-fold, respectively, using dichloromethane in accordance with the method described by Schieberle [34]. For each odor-active compound, the flavor dilution (FD) factor was uncovered as the highest dilution level where the odor can still be identified by the sensory panel during GC–O–MS analysis. To ensure reliability, each panelist performed three replicate analyses for every extract [12].

### 2.6. Quantification of Aroma Compounds

The aroma components 2-methylphenol, 2-hydroxy-3-methyl-2-cyclopentenone, 3-phenylpropionic acid, 2-methylpropionic acid, 4-hydroxy-2,5-dimethylfuran-3-one, 2-methoxy-4-vinylphenol, butyric acid, pentanoic acid, 2-methyl-1-propanol, 3-methyl-2-cyclopenten-1-one, Furan-2,5-dicarbaldehyde, 2,6-diethylpyrazine, 1*H*-pyrrole-2-carbaldehyde, and 3-phenylpyridine were determined quantitatively following SPE with a LiChrolut EN cartridge. Initially, 20.0 mL of JMHJ was mixed with 3.0 g sodium chloride and 5.0 μL of an ethanolic internal standard mixture consisting of 4-octanol (1000 mg/L), cinnamyl acetate (1000 mg/L), amyl acetate (1000 mg/L), 2-ethylbutanoic acid (5000 mg/L), and ethyl maltol (1000 mg/L). Subsequently, the extraction process was performed according to the above method. After filtration, the eluates were dehydrated with anhydrous sodium sulfate, reduced to a final volume of 0.20 mL with a gentle stream of high-purity nitrogen, and maintained at −40 °C prior to analysis [12].

Quantitative analysis of the other compounds was conducted employing SPME in combination with GC–MS [10]. A 5.0 mL aliquot of JMHJ, supplemented with 5.00 μL of an internal standard mixture containing 4-octanol (1000 mg/L), cinnamyl acetate (1000 mg/L), amyl acetate (1000 mg/L), and 2-ethylbutanoic acid (5000 mg/L), was subjected to SPME extraction following the procedure described above.

Quantification was performed using 12 concentration levels of either mixed or single-compound standard solutions prepared in 11.5% (*v*/*v*) ethanol–water. Following the addition of the corresponding internal standards, these solutions were subjected to the same analytical procedure as that used for the relevant samples. All determinations were conducted three times. Calibration curves based on the internal standard method were constructed from plots of the peak response ratio of each analyte to that of its internal standard against the corresponding concentration ratio. The regression equations for the individual calibration lines are listed in Table 2 [12].

The quantification of the aroma-active compounds was performed by GC–MS with a Trace 1310 gas chromatograph linked to an ISQ mass spectrometer (Thermo Fisher Scientific, Waltham, MA, USA). Helium (99.999%) was employed as the carrier gas at 1.0 mL/min. Samples of 1.0 μL were introduced in a split mode (20:1) with the injector maintained at 250 °C. Separation was conducted on a DB-WAX column (30 m × 0.25 mm × 0.25 μm; J&W Scientific, Folsom, CA, USA). The oven temperature was first held at 40 °C, and then increased to 50 °C at 2.0 °C/min and kept for 5.0 min, followed by heating to 80 °C at 3.0 °C/min with a further 5.0 min hold, and eventually raised to 240 °C at 5.0 °C/min and kept for 10 min. Detection was performed in electron impact ionization mode at 70 eV. The transfer line, ion source, and quadrupole temperatures were maintained at 250, 240, and 150 °C, respectively. Quantitative analysis of all target compounds was conducted in selected ion monitoring (SIM) mode. Detailed information on the qualifier ions, quantifier ions, and calibration equations is presented in Table A2 [12].

### 2.7. Calculation of Odor Activity Value (OAV)

OAVs for the identified aroma compounds were determined by dividing the concentration of each compound by its respective odor threshold.

### 2.8. Aroma Reconstitution Experiments

To confirm the significance and aroma contribution of odorants in JMHJ, compounds with relatively high OAVs (≥1) were introduced to the JM-T matrix at the concentrations detected in the samples. The prepared model systems were subsequently subjected to sensory evaluation according to the method outlined in Section 2.2. The JM-T matrix was prepared as follows: Briefly, a 20 mL JM-T sample was loaded onto an SPE column that had been preconditioned sequentially with 6.0 mL of dichloromethane, methanol, and ultrapure water. The loading flow rate was maintained below 2 mL/min. After sample passage, the column was washed with 6.0 mL of ultrapure water. The fraction eluted with water was collected, freeze-dried, and subsequently dissolved in 20 mL of an 11.5% (*v*/*v*) ethanol/water solution.

### 2.9. Addition Experiments

To verify the compounds that played vital roles in the aroma of JMHJ, addition tests were carried out [12,35]. Specifically, authentic standards were first dissolved in a 11.5% (*v*/*v*) aqueous ethanol solution to prepare spiking solutions. Compound spiking was performed using a low-level JMHJ sample as the base matrix. For each target compound, the amount added was calculated so that the final concentration in the spiked sample matched the concentration measured in a high-level JMHJ sample (i.e., a “low-to-high” matching design). After they were added, samples were mixed thoroughly and allowed to equilibrate before sensory evaluation.

For the addition experiments, one or several odorants were introduced into JMHJ to prepare spiked samples, which were then subjected to triangle testing [36]. In the triangle test, 10.0 mL of each addition model was assessed in comparison with two untreated JMHJ samples. The statistical significance of the perceptible differences was evaluated following the procedure described by Lawless [37]. The addition tests were carried out by the same sensory panel as that introduced in Section 2.2.

### 2.10. Statistical Analysis

All graphical illustrations were generated employing OriginPro 9.0. Statistical analyses, including Duncan’s multiple range test (*p* ≤ 0.05), were carried out utilizing SPSS 22.0 (SPSS Inc., Chicago, IL, USA). Pearson’s two-tailed correlation test was employed to assess the similarity between the original samples and their respective recombination models.

## 3. Results and Discussion

### 3.1. Sensory Evaluation Results

**Figure 1:**
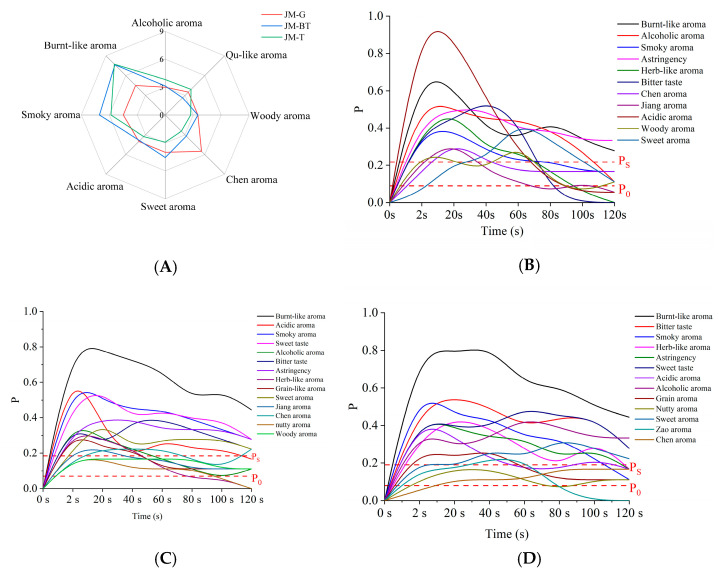
(**A**) Orthonasal aroma profile of Jimo Huangjiu. Aroma descriptors shown here were obtained from orthonasal evaluation (before ingestion). (**B**–**D**) Dynamic sensory perception during consumption of Jimo Huangjiu. P_0_ is the presented chance level: the dominance rate that an attribute may reach randomly. P_S_ is the significance level, defined as the minimum proportion required for the dominance rate to be considered significantly higher than P_0_.

As illustrated in Figure 1A, JMHJ’s aroma profile includes smoky, burnt-like, alcoholic, Qu-like, woody, Chen, sweet, and acidic aromas. The intensities of the smoky and burnt-like aromas were the highest. This distinguishes JMHJ markedly from other types of Huangjiu. For instance, traditional Huangjius of Shaoxing/Shanghai are characterized by herb-like, Qu-like, and grain aromas [13,14], whereas coarse-grain Huangjiu emphasizes cooked grain, acidic, and alcoholic aromas [12].

Beyond the orthonasal aroma that influences initial consumer choice, the retronasal aroma experienced during consumption contributes equally to consumer preference. To elucidate this dynamic sensory experience, TDS analysis was employed to characterize the evolution of flavor perception during JMHJ consumption. JM-G exhibited 11 distinct flavor characteristics with temporal dominance patterns during consumption: acidic taste dominated initially (0–42 s), followed sequentially by bitter taste (42–55 s), alcoholic aroma (58–68 s), burnt-like aroma (68–100 s), and astringency (100–120 s) (Figure 1B). JM-BT presented 14 flavor characteristics, with burnt-like aroma being the dominant attribute (dominance probability > 0.6), accompanied by three strongly perceived characteristics of acidic, smoky, and sweet aromas (Figure 1C). JM-T displayed 13 flavor characteristics, with burnt-like aroma having an even stronger dominance (probability > 0.8), alongside prominent attributes of acidic, smoky, and sweet aromas (Figure 1D).

Notably, despite variations in sweetness level (taste) and temporal patterns, the burnt-like aroma consistently dominated the retronasal olfaction profile across all three JMHJs during oral consumption, confirming its role as the iconic sensory characteristic of JMHJ. The consistent dominance of the burnt-like aroma during consumption underscores its importance in defining the unique sensory identity of JMHJ, highlighting the necessity of a comprehensive investigation into aroma perception mechanisms to optimize product quality and understand consumer acceptance.

### 3.2. Identification of Aroma-Active Compounds

As shown in Table 1, 68 odorants were detected in JMHJ via GC-O-MS, encompassing 16 nitrogen-containing compounds (NCCs), 12 esters, 9 ketones, 9 acids, 9 furans, 5 phenols, 3 alcohols, 1 sulfur-containing compound, 2 acetals, and 2 aldehydes. Notably, 13 of the identified compounds were newly recognized as aroma-active substances in Huangjiu, including 3-methyl-2-cyclopenten-1-one (sweet, fruity, and woody, AI = 2, and FD > 10); 2-hydroxycyclopent-2-en-1-one (caramel, AI = 2, and FD > 10); 4-phenyl-2-butanone (floral and fat, AI = 5, and FD > 100); 2-ethyl-3-methylpyrazine (green, roasted, nuts, with roasted and smoky characteristics, AI = 5, and FD > 100); 2-ethylpyrazine (roasted, woody with meaty and savory characteristics, AI = 2, and FD > 10); 2,3-dimethylpyrazine (roasted, cocoa, featuring meaty, savory, milky, smoky notes, AI = 2, and FD > 10); 2-ethyl-6-methylpyrazine (roasted nuts, characterized by a roasted aroma, AI = 2, and FD > 10); 2,6-diethylpyrazine (nuts, AI = 2, and FD > 10); 2-acetylpyridine (popcorn, roasted nut, AI = 2, and FD > 1); 5-methyl-2-acetylpyrazine (popcorn, AI = 2, and FD > 1); 1*H*-pyrrole-2-carbaldehyde (roasted and coffee, AI = 5, and FD > 100); 3-phenylpyridin (medicine, AI = 1, and FD > 10); and 2-phenylethylpyrazine (nuts, AI = 1, and FD > 1).

Esters are generally considered the most abundant odorants in fermented beverages, such as coarse cereal Huangjius, Shaoxing Huangjiu, Daixian Huangjiu, wine, baijiu, and beer [25]. However, this study revealed that the NCCs were the dominant class of aroma-active compounds of JMHJ. The Maillard reaction, which occurs during prolonged high-temperature reactions in the millet boiling process, may be the main reason for the abundance of pyrazine compounds [23,24]. A total of seven NCCs were detected in the boiled millet according to a previous study by Jiang [5], including 2-methylpyrazine (nuts, woody, characterized by soy sauce-like and savory notes, AI = 2, and FD > 10); 2,6-dimethylpyrazine (sunflower seeds, characterized by savory and vegetable-like aroma, AI = 2, and FD > 10); 2-ethylpyrazine (roasted, woody with meaty and savory characteristics, AI = 2, and FD > 10); 2,3-dimethylpyrazine (roasted, cocoa, featuring meaty, savory, milky, and smoky, AI = 2, and FD > 10); 2-ethyl-6-methylpyrazine (roasted nuts, characterized by a roasted aroma, AI = 2, and FD > 10); 2-ethyl-3-methylpyrazine (green, roasted, nuts, with roasted and smoky characteristics, AI = 5, and FD > 100); 2,6-diethylpyrazine (nuts, AI = 2, and FD > 10); and 2-acetyl-1*H*-pyrrole (sunflower seed, AI = 2, and FD > 10). It was suggested that these compounds may originate from the materials used; however, this observation alone does not directly transfer into findings and would require process-tracking evidence for confirmation. In addition, among all 16 NCCs above, 10 of them were identified in Huangjiu for the first time, including seven pyrazines (which mainly exhibit a roasted nut odor), as well as 2-acetopyridines (with popcorn and roasted nut aromas), 1*H*-pyrrole-2-carboxaldehyde (roasted coffee, AI = 5, and FD > 100), and 3-phenylpyridine (medicine, AI = 1, and FD > 10).

Ketones were the second most abundant odorants in JMHJ; they were found to mainly exhibit a woody-like aroma, such as minty, mushroom, and maple odor. 1-(2-Methylcyclopenten-1-yl)ethanone (woody, AI = 2, FD > 10); 2-hydroxycyclopent -2-en-1-one (maple, caramel, AI = 2, FD > 10); and 4-phenyl-2-butanone (floral, balsam, AI = 5, FD > 100) were newly detected Huangjiu. Additionally, 2-hydroxy-3-methyl-2-cyclopentenone (caramel), 4-phenyl-2-butanone (floral balsam), and 4-hydroxy-2,5-dimethylfuran -3-one (sotolone) (caramel, herbal) had the strongest aromatic intensities (AI = 5, FD > 100) in JMHJ. Among them, 2-hydroxy-3-methyl-2-cyclopentenone was detected in Shaoxing Huangjiu [38] and sotolone was identified as the key odorant in Huangjiu in previous studies [10]. By contrast, 4-phenyl-2-butanone was newly distinguished in Huangjiu in this investigation.

Esters constituted the third largest group of odorants and were primarily generated either through esterase-mediated esterification between acids and alcohols derived from amino acids and glucose during microbial metabolism, or through non-enzymatic reactions occurring between organic acids and alcohols [25]. Among them, ethyl phenylacetate (floral and sweet, AI = 5, FD > 100) and ethyl 3-phenylpropanoate (sweet, wine, floral, AI = 4, FD > 100) were the strongest odors in JMHJ, and are ubiquitously found in most varieties of Huangjiu [10].

Acids contributed to acidic, cheesy, and sour aromas, including butyric acid, 2-methylpropionic acid, acetic acid, 3-methylbutanoic acid, 3-phenylpropionic acid, decanoic acid, nonanoic acid, octanoic acid, hexanoic acid, and pentanoic acid. The AI of all acidic compounds is moderate (AI = 3, FD > 10). The formation of these compounds was mainly associated with fatty acid metabolic pathways and with oxidative reactions involving alcohols and aldehydes [26]. Moreover, these acids were previously reported in Daixian Huangjiu [10], Shaoxing Huangjiu [39], and cereal Huanjgiu [12].

A total of five phenolic compounds exhibiting smoky, spicy, and woody sensory attributes were detected in JMHJ, i.e., 2-methoxyphenol (smoke, sweet, and medicine); 2-methylphenol (leather); 4-ethyl-2-methoxyphenol (clove); 4-ethylphenol (dry soil and animals), and 2-methoxy-4-vinylphenol (cloves and curry). The aroma intensities of these compounds were consistently rated as high (AI = 5, FD > 100). The formation of these compounds was primarily attributed to the decarboxylation of hydroxycinnamic acid derivatives, especially p-coumaric acid and ferulic acid [27]. It was noteworthy that all phenolic compounds exhibited intense odors that remained perceptible even after 100-fold dilution. Phenols also existed in other Huangjiu, but their aroma intensity was generally weaker [17,39]. It was indicated that phenolic substances were particularly prominent in JMHJ, reflecting its important aromatic characteristics.

In addition, three alcohols, two aldehydes, two acetals, and one sulfur-containing compound were identified in JMHJ. In alcoholic beverages, alcohols are generally regarded as an important group of odorants and are mainly formed through sugar metabolism as well as the dehydrogenation and decarboxylation of amino acids for the period of fermentation [28]. However, only 2-methyl-1-propanol (fusel and fruity, AI = 2, FD > 10); 3-methyl-1-butanol (fusel, fruity, AI = 2, FD > 10); 2-furanmethanol (Burnt, AI = 2, FD > 100); and *β*-phenethyl alcohol (rose, AI = 5, FD > 100) were distinguished by GC-O-MS in JMHJ. *β*-phenethyl alcohol, 3-methyl-1-butanol, and 2-Methyl-1-propanol were primarily produced through the metabolism of Leucine, Valine, and phenylalanine, respectively [27]. The formation of 2-furanmethanol is closely associated with the Maillard reaction, the pyrolysis of sugars, and chemical reactions during fermentation. Aldehydes in Huangjiu were produced during the fermentation process, mostly generated through the deamination and decarboxylation of amino acids [40].

Given the multifactorial nature of Huangjiu processing, the origins of aroma-active compounds in JMHJ cannot be uniquely attributed to a single pathway but rather reflect the combined effects of raw-material carryover, thermal reactions, and fermentation-driven formation/transformation. Many esters, higher alcohols, and volatile acids are plausibly related to microbial fermentation and subsequent esterification/metabolic conversions [25,26,27,28]; on the other hand, nitrogen-containing heterocycles (e.g., pyrazines, pyrroles, and pyridines) and several furanic/cyclopentenone-type compounds are more consistent with formation or enrichment via Maillard/Strecker-type chemistry [23,24,41]. These compounds may undergo further transformation during fermentation and subsequent vinification/aging, e.g., microbial amino-acid metabolism, redox reactions, and matrix interactions [23,24].

### 3.3. Quantification Analysis

**Figure 2:**
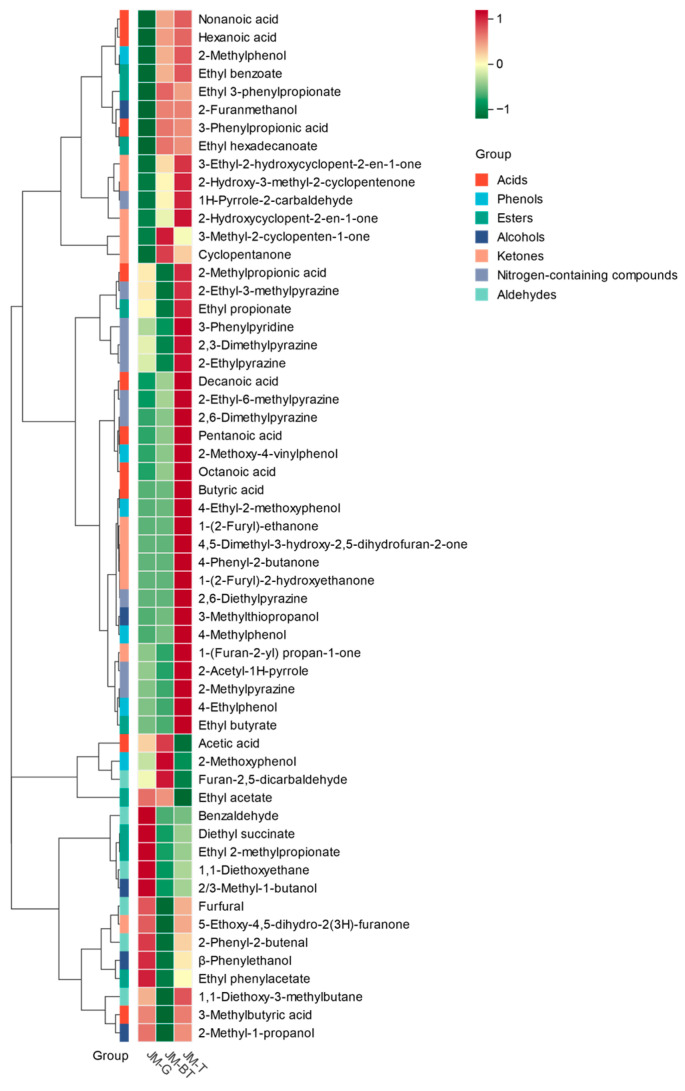
Heatmap of the concentrations of aroma-active compounds in Jimo Huangjiu. Concentration data were row-wise z-score standardized (by sample) prior to plotting.

For a more detailed comparison of the aroma profiles of the three JMHJ samples, quantitative analysis was carry out on 57 odor-active compounds with FD values exceeding 10. Their concentration data are illustrated in Table 2 and graphically presented in Figure 2. In addition, 15 aroma-active compounds were newly quantified in Huangjiu. These comprised 2-ethyl-6-methylpyrazine (534.39 μg/L), 2-ethyl-3-methylpyrazine (28.95 μg/L), and cyclopentanone (48.06 μg/L).

3-Methylbutanoic acid (60,501.13 μg/L), ethyl acetate (38,742.47 μg/L), *β*-phenethyl alcohol (29,306.76 μg/L), and 1,1-diethoxyethane (16,600.20 μg/L) had the highest average concentrations in JMHJ. In particular, the concentration of 3-methylbutanoic acid in JMHJ was drastically higher compared with other Huangjius, such as Shaoxing Huangjiu (3100 μg/L) [13] and Daixian Huangjiu (849.43 μg/L) [10]. The concentrations of ethyl acetate, *β*-phenethyl alcohol, and 1,1-diethoxyethane in JMHJ were higher than those in coarse cereal Huangjiu (17,000 μg/L, 21,100 μg/L, and 282 μg/L, respectively) [12], but lower than concentrations in Shaoxing Huangjiu (83,970 μg/L, 109,310 μg/L, and 137,500 μg/L, respectively) [39].

Among all categories of odorants, acids had the highest total concentrations, followed by esters and alcohols. The highest total concentration of acids was mainly due to the high concentration of 3-methylbutanoic acid (60,501.13 μg/L). This contradicts the commonly held view that esters and alcohols are the predominant compounds in fermented beverages. JM-BT exhibited lower total concentrations of acids, alcohols, and acetals (21,229.60, 21,597.97, and 11,504.84 μg/L, respectively) compared to JM-G (83,528.41, 43,459.04, and 21,803.88 μg/L) and JM-T (86,010.90, 34,472.94, and 14,215.08 μg/L). The concentration of ester was the highest in JM-G (52,807.45 μg/L) and the lowest in JM-T (34,662.52 μg/L), while the concentration of aldehydes in JM-G (3329.49 μg/L) was markedly higher than that in JM-BT (1393.30 μg/L) and JM-T (1712.69 μg/L). Conversely, JM-T contained substantially higher levels of NCCs, ketones, phenols, and sulfur-containing compounds (4029.78, 3327.56, 2252.57, and 10.44 μg/L) than those in JM-G (1512.40, 1122.20, 287.29, and 13.36 μg/L) and JM-BT (1694.80, 1731.04, 420.90, and 62.88 μg/L).

According to the GC-O results, the nitrogen-containing and phenolic compounds were characteristic of JMHJ. Among all the NCCs, 2,6-dimethylpyrazine was detected at a relatively high concentration in the analyzed JMHJ samples, with measured values of 808.22 μg/L in JM-G, 939.30 μg/L in JM-BT, and 1795.67 μg/L in JM-T. The concentration of 2,6-dimethylpyrazine was significantly higher in JMHJ than in Shaoxing Huangjiu, where it was only 19.50 μg/L [14,39]. Similarly, 61.59 μg/L of 2-methylpyrazine was found in JMHJ, compared to only 9.2 μg/L in Shaoxing Huangjiu [9]. Additionally, these six NCCs were quantified in Huangjiu for the first time, including 2-acetyl-1*H*-pyrrole (744.03 μg/L), 2-ethyl-6-methylpyrazine (534.39 μg/L), 2-ethyl-3-methylpyrazine (28.95 μg/L), 2,3-dimethylpyrazine (21.48 μg/L), 2-ethylpyrazine (10.74 μg/L), and 2,6-diethylpyrazine (0.61 μg/L).

The concentration of phenolic compounds in JMHJ was greater than in other types of Huangjiu. Among these, 4-ethyl-2-methoxyphenol had the highest concentration (669.20 μg/L), with levels significantly greater than the concentration in Shaoxing Huangjiu (10.3–101 μg/L) [14,39]. The concentration of 4-methylphenol (216.15 μg/L) was also higher than that in Shaoxing Huangjiu (1.9 μg/L) [39]. Additionally, the level of 4-ethylphenol in JMHJ (35.18 μg/L) surpassed that in Daixian Huanjgiu (18.17 μg/L) [10], coarse cereal Huangjiu (1.48 μg/L) [12], and Shaoxing Huangjiu (27.7 μg/L) [14]. 2-methylphenol (17.64 μg/L) was quantified in Huangjiu for the first time.

The concentration of aldehydes was lower in Shaoxing Huangjiu. For example, benzaldehyde (1430.31 μg/L) possessed the greatest concentration in JMHJ compared to 5250 μg/L in Shaoxing Huangjiu [39]. The concentrations of furfural (468.92 μg/L) and 2-phenyl-2-butenal (235.73 μg/L) were also lower in JMHJ than in Shaoxing Huangjiu, at 6250 μg/L and 199 μg/L, respectively [39]. Ketones were abundant in JMHJ, and this investigation quantified a total of 11 compounds. It is noteworthy that all these compounds were identified and quantified in Huangjiu for the first time. 3-Methyl-2(5H)-furanone (644.55 μg/L), 1-(furan-2-yl) propan-1-one (296.05 μg/L), and 4-hydroxy-2,5-dimethylfuran-3-one (189.89 μg/L) were the most abundant ketones in JMHJ.

Fusel alcohols, together with branched-chain acids including 3-methylbutanoic acid, are plausibly linked through amino-acid catabolism-related pathways during fermentation (e.g., branched-chain amino acids yield higher corresponding alcohols and acids); however, multiple formation and transformation routes may coexist in Huangjiu [21]. A high level of 3-methylbutanoic acid was observed in JMHJ (60,501.13 μg/L). In comparison, the mean concentrations of related fusel alcohols were 2608.92 μg/L for 2-methyl-1-propanol and 1065.91 μg/L for 2/3-methyl-1-butanol. Shaoxing and Daixian Huangjiu are described to contain much higher levels of 3-methyl-1-butanol (214,200 and 114,664.63 μg/L, respectively) than 3-methylbutanoic acid (3100 and 849.43 μg/L, respectively). In contrast, JMHJ had the opposite pattern, with 3-methylbutanoic acid far exceeding the corresponding fusel alcohol [10,39]. This contrast suggests there are region- and process-dependent differences in precursor availability, microbial activity, and the balance of downstream oxidation/esterification.

### 3.4. Calculation of OAVs

The aroma contribution of volatile constituents in JMHJ was evaluated by estimating the OAVs of the identified odor-active compounds using odor threshold values reported in previous studies. As shown in Table 3, 26, 27, and 27 odorants exhibited OAVs ≥ 1 in JM-G, JM-BT, and JM-T, respectively. Ethyl 2-methylpropionate had the highest average OAV of 94,960,667, followed by 4-methylphenol (OAV = 27, 019, and 167; the latter had the same OAV as this), *β*-phenethyl alcohol (14,653,380), 2-ethyl-6-methylpyrazine (8,906,500), 4-ethyl-2-methoxyphenol (3,346,000), 2-methylpyrazine (1,026,500), 2-methoxyphenol (1,017,556), and 2-methylphenol (569,032).

These odorants with OAV > 1 included nine esters, six NCCs, five phenols, four acids, two ketones, one alcohol, one sulfur-containing compound, one aldehyde, one furan, and one acetal. Among the nine esters identified, ethyl 2-methylpropionate (94,960,667), ethyl hexadecanoate (61,222), and ethyl 3-phenylpropanoate (184) had the highest OAVs. This was the first time that the importance of ethyl 2-methylpropionate and ethyl hexadecanoate in the aroma of Huangjiu had been identified. 3-Phenylpropanoate was recognized as an essential odorant in Daixian Huangjiu in a earlier investigation [10].

**Figure 3:**
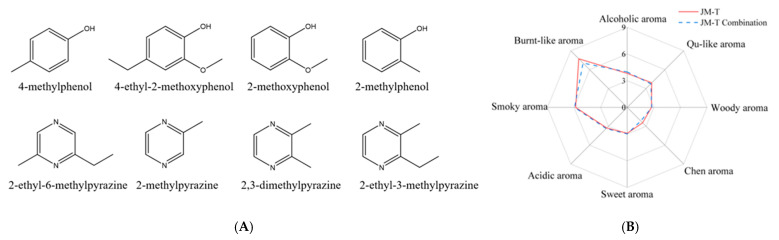
Chemical structures of key odorants contributing to smoky and burnt-like notes (**A**); the recombination results of JMHJ (**B**).

It was also detected that among the six NCCs, five had an average OAV >2000, namely, 2-ethyl-6-methylpyrazine (8,906,500), 2-methylpyrazine (1,026,500), 2,3-dimethylpyrazine (268,458), 2-ethyl-3-methylpyrazine (222,667), and 2-ethylpyrazine (2685). Additionally, four out of five phenols with an average OAV >1 had a relatively high OAV (>500,000), including 4-methylphenol (27,019,167), 4-ethyl-2-methoxyphenol (3,346,000), 2-methoxyphenol (1,017,556), and 2-methylphenol (569,032). Pyrazine compounds were mainly associated with the aroma of roasted nuts, whereas phenolic compounds mainly exhibited a smoky aroma. Their chemical structures are depicted in Figure 3A. Taken together, these results indicate that both NCCs and phenols are key contributors to the JMHJ aroma.

### 3.5. Aroma Recombination and Addition

Compounds exhibiting OAVs above 1 in JM-T were selected for aroma recombination experiments. The results, exhibited in Figure 3B, indicate that the aroma profile of the recombined sample closely resembles that of the original sample, with a similarity of 94.5%.

To confirm the influence of phenolic and pyrazine compounds to the JMHJ aroma, these compounds were individually added to the original samples for sensory evaluation. The original samples contained relatively low concentrations of these compounds before addition. For example, 2-methoxyphenol with a lower concentration in JM-T was added to the JM-T until its concentration matched that in the JM-G (Addition group 1 in Table 4). Similarly, 4-methylphenol, 2-methylphenol, 4-ethyl-2-methoxyphenol, and 2-methoxy-4-vinylphenol were added to the JM-G to achieve the concentrations as JM-T (Addition group 2 in Table 4). The sensory assessment outcomes are shown in Table 4. The addition of the six phenolic compounds enhanced the smoky aroma of the original samples. In addition, except for 4-ethyl-2-methoxyphenol, all phenolic compounds intensified the burnt-like aroma of the original samples, while 2-methoxyphenol and 4-methylphenol enhanced the sweet aroma of the samples.

Pyrazine compounds exhibited diverse aromatic characteristics and contributed differentially to the aroma of Huangjiu. 2-Methylpyrazine, characterized by soy sauce-like and savory notes, enhanced sweet, burnt-like, and acidic aromas. 2-Ethylpyrazine (meaty and savory) strengthened woody and smoky notes, while 2,3-dimethylpyrazine (meaty, savory, milky, and smoky) intensified smoky and roasted aromas. 2,6-Dimethylpyrazine (savory and vegetable-like) further enhanced the smoky aroma, whereas 2-ethyl-3-methylpyrazine (roasted and smoky) contributed to sweet and smoky notes. Finally, 2-Ethyl-6-methylpyrazine (roasted) primarily enhanced woody and sweet aromas. Collectively, most pyrazines predominantly contributed to the burnt-like, smoky, and roasted aroma profile of Huangjiu (Table 4).

Addition experiments confirmed the essential contribution of pyrazines and phenolic compounds to the burnt-like and smoky aroma of JMHJ, consistent with the quantitative and sensory results. JM-T had elevated levels of these compounds, and correspondingly, greater intensity and persistence of burnt-like and smoky aromas were detected in both orthonasal and retronasal olfaction compared to JM-G and JM-BT. These observations support the conclusions drawn from this study.

In addition, GC-O analysis revealed that the elution regions of 4-ethylphenol and nonanoic acid were adjacent, exhibiting a strong Qu-like aroma. However, when these two compounds were evaluated individually via olfaction, neither exhibited Qu-like aroma characteristics. To investigate the mechanism underlying the formation of Qu-like aroma, 4-ethylphenol and nonanoic acid were added separately and in combination to Huangjiu samples for sensory evaluation (Addition group 3 in Table 4). The outcomes revealed that the addition of 4-ethylphenol alone did not enhance Qu-like aroma. By contrast, the addition of nonanoic acid significantly enhanced this attribute when added individually or in combination with 4-ethylphenol (Table 4). Considering the fact that the original Huangjiu samples already contained high levels of phenolic compounds, these findings suggest that nonanoic acid contributes to the formation of a Qu-like aroma through synergistic interactions with endogenous phenolic substances present in Huangjiu, rather than acting independently. Therefore, nonanoic acid is recognized as an essential contributor to Qu-like aroma in Huangjiu, functioning not as an independent aroma compound but as a synergistic partner with endogenous phenolic substances that generate this characteristic sensory attribute.

The Qu-like aroma, among others, is the most characteristic sensory characteristic of Chinese Huangjiu and Baijiu. Qu, serving as both a saccharification and fermentation starter, imparts this distinctive aroma to alcoholic beverages through complex microbial fermentation processes. These aroma characteristics have an essential role in determining product quality and consumer acceptance. However, the specific volatile compounds and mechanisms responsible for the formation of Qu-like aroma remain incompletely understood. The present study demonstrates, for the first time, that the synergistic interactions between nonanoic acid and phenolic compounds contribute to the generation of Qu-like aroma, providing valuable insights for quality control and aroma optimization in Huangjiu production. However, the detailed molecular mechanisms underlying these interactions require further investigation.

## 4. Conclusions

In this paper, we characterized the aroma characteristics of JMHJ through both static sensory evaluation (before drinking) and dynamic sensory evaluation (during consumption). Burnt-like and smoky aromas are characteristic of JMHJ. Using SPE and SPME combined with GC-O-MS, 68 aroma-active compounds were classified. Thirteen of these compounds were newly recognized as aroma-active compounds in Huangjiu, fifteen were first quantified in Huangjiu, and twenty-eight aroma compounds with OAVs greater than one were identified in JMHJ. The compounds with the highest OAVs included ethyl 2-methylpropionate (94,960,667), 4-methylphenol (27,019,167), *β*-phenylethyl alcohol (14,653,380), 2-ethyl-6-methylpyrazine (8,906,500), 4-ethyl-2-methoxyphenol (3,346,000), 2-methylpyrazine (1,026,500), 2-methoxyphenol (1,017,556), 2-methylphenol (569,032), 2-hydroxy-3-methyl-2-cyclopentenone (498,911), 2,3-dimethylpyrazine (268,458), and 2-ethyl-3-methylpyrazine (222,667).

Additionally, the concentrations of pyrazines and phenolic compounds in JMHJ were significantly higher compared with those of other Huangjiu varieties. These compounds were identified as characteristic markers of JMHJ. Addition experiments confirmed that pyrazines and phenolic compounds contributed to the smoky and burnt-like aroma characteristics of JMHJ. Moreover, the interaction between nonanoic acid and phenolic compounds was found to enhance the Qu-like aroma of Huangjiu. These new findings provide an important reference value for controlling and optimizing the flavor quality of JMHJ.

## Figures and Tables

**Table 1:** Identified odor-active compounds in Jimo Huangjiu.

No.	Aroma-Active Compounds	Formula	Odor Descriptors	Aroma Intensity	FD Factor	cRI/lRI ^b^		Identification ^c^
	**Esters**							
1	Ethyl acetate	C_4_H_8_O_2_	Ester	1	>10	870/878	615/610	MS, RI, aroma, S
2	Ethyl propionate	C_5_H_10_O_2_	Fruity	1	>10	938/947	728/726	MS, RI, aroma, S
3	Ethyl 2-methylpropionate	C_6_H_12_O_2_	Fruity	2	>10	983/971	769/747	MS, RI, aroma, S
4	Ethyl butyrate	C_6_H_12_O_2_	Fruity	1	>10	1026/1036	809/799	MS, RI, aroma, S
5	Ethyl 3-methylbutyrate	C_7_H_14_O_2_	Fruity	2	>1	1078/1067	855/854	MS, RI, aroma, S
6	Ethyl 2-hydroxy-3-methylbutyrate	C_7_H_14_O_3_	Fruity	2	>1	1426/1422	997/975	MS, RI, aroma, S
7	Ethyl benzoate	C_9_H_10_O_2_	Sweet, floral	3	>10	1644/1658	1188/1171	MS, RI, aroma, S
8	Diethyl succinate	C_8_H_14_O_4_	Fruity	3	>10	1691/1677	1195/1179	MS, RI, aroma, S
9	Ethyl phenylacetate	C_10_H_12_O_2_	Floral, sweet	5	>100	1813/1782	1224/1229	MS, RI, aroma, S
10	Ethyl 3-phenylpropionate	C_11_H_14_O_2_	Sweet, wine, floral	4	>100	1889/1900	1342/1324	MS, RI, aroma, S
11	Diethyl malate	C_8_H_14_O_5_	Sweet	5	>1	2065/2047	1282/1270	MS, RI, aroma, S
12	Ethyl hexadecanoate	C_18_H_36_O_2_	Wax	3	>10	2246/2243	1986/1994	MS, RI, aroma, S
	**Alcohols**							
13	2-Methyl-1-propanol	C_4_H_10_O	Fusel, fruity	2	>10	1108/1108	602/622	MS, RI, aroma, S
14	2/3-Methyl-1-butanol	C_5_H_10_O	Fusel, fruity	2	>10	1185/1200	727/720	MS, RI, aroma, S
15	2-Furanmethanol	C_5_H_6_O_2_	Burnt odor	5	>100	1640/1635	866/845	MS, RI, aroma, S
16	*β*-Phenylethanol	C_8_H_10_O	Rose	5	>100	1942/1902	1117/1116	MS, RI, aroma, S
	**Acids**							
17	Acetic acid	C_2_H_4_O_2_	Sour	3	>10	1453/1441	589/600	MS, RI, aroma, S
18	2-Methylpropionic acid	C_4_H_8_O_2_	Sour	3	>10	1581/1556	807/790	MS, RI, aroma, S
19	Butyric acid	C_4_H_8_O_2_	Sour, sweaty	3	>10	1637/1628	873/848	MS, RI, aroma, S
20	3-Methylbutyric acid	C_5_H_10_O_2_	Sweat	3	>10	1680/1661	892/875	MS, RI, aroma, S
21	Pentanoic acid	C_5_H_10_O_2_	Sweat, putrid	3	>10	1720/1713	946/933	MS, RI, aroma, S
22	Hexanoic acid	C_6_H_12_O_2_	Sweat, cheese	3	>10	1882/1866	992/981	MS, RI, aroma, S
23	Octanoic acid	C_8_H_16_O_2_	Putrid, sour	2	>10	2129/2072	1199/1191	MS, RI, aroma, S
24	Nonanoic acid	C_9_H_18_O_2_	Cheese	3	>10	2150/2144	1280/1278	MS, RI, aroma, S
25	Decanoic acid	C_10_H_11_NO_6_	Putrid	3	>10	2320/2365	1390/1380	MS, RI, aroma, S
26	3-Phenylpropionic acid	C_9_H_10_O_2_	Salivary, sweet	3	>10	2580/2603	1341/1344	MS, RI, aroma, S
	**Aldehydes**							
27	Furfural	C_5_H_4_O_2_	Roasted	2	>10	1478/1464	822/848	MS, RI, aroma, S
28	Benzaldehyde	C_7_H_6_O	Roasted, fruity	2	>10	1544/1530	913/921	MS, RI, aroma, S
29	2-Phenyl-2-butenal	C_10_H_10_O	Green, vegetables, floral, nuts	1	>10	1933/1907	1269/1281	MS, RI, aroma, S
30	Furan-2,5-dicarbaldehyde	C_6_H_4_O_3_	Sweet, caramel	5	>100	2014/1986	1078/1076	MS, RI, aroma, S
	**Ketones**							
31	Cyclopentanone	C_5_H_8_O	Peppermint	2	>10	1126/1144	800/808	MS, RI, aroma, S
32	3-Hydroxybutanone	C_4_H_8_O_2_	Yogurt	2	>1	1296/1280	709/718	MS, RI, aroma, S
33	1-Octen-3-one	C_8_H_14_O	Mushroom	3	>1	1308/1317	983/976	MS, RI, aroma, S
34	1-(2-Methylcyclopent-1-en-1-yl)ethanone	C_8_H_12_O	Woody	2	>1	1495/1504		MS, RI, aroma, S
35	1-(2-Furyl)-ethanone	C_6_H_6_O_2_	Woody, roasted	2	>10	1512/1499	896/910	MS, RI, aroma, S
36	3-Methyl-2-cyclopenten-1-one ^a^	C_6_H_8_O	Sweet, fruity, woody	2	>10	1518/1513	956/976	MS, RI, aroma
37	1-(Furan-2-yl) propan-1-one	C_7_H_8_O_2_	Burned, rubbery	2	>10	1563/1565	984/1008	MS, RI, aroma
38	2-Hydroxycyclopent-2-en-1-one ^a^	C_5_H_6_O_2_	Caramel	2	>10	1749/1769	901/926	MS, RI, aroma
39	2-Hydroxy-3-methyl-2-cyclopentenone	C_6_H_8_O_2_	Caramel	5	>100	1824/1830	1022/1037	MS, RI, aroma, S
40	4-Phenyl-2-butanone ^a^	C_10_H_12_O	Floral, fat	5	>100	1848/1858	1241/1218	MS, RI, aroma, S
41	3-Ethyl-2-hydroxycyclopent-2-en-1-one	C_7_H_10_O_2_	Savory	4	>100	1937/1924	1125/1140	MS, RI, aroma
42	1-(2-Furyl)-2-hydroxyethanone	C_6_H_6_O_3_	Roasted	5	>100	2019/2000	1054/1070	MS, RI, aroma
43	1,1-Diethoxyethane	C_6_H_14_O_2_	Fruity	2	>10	894/892	731/725	MS, RI, aroma, S
44	1,1-Diethoxy-3-methylbutane	C_9_H_20_O_2_	Green	2	>10	1061/1065	939/955	MS, RI, aroma, S
45	5-Ethoxy-4,5-dihydro-2(3*H*)-furanone	C_6_H_10_O_3_	Burnt	2	>10	1722/1728	1064/1067	MS, RI, aroma
46	4,5-Dimethyl-3-hydroxy-2,5-dihydrofuran-2-one	C_6_H_8_O_3_	Caramel, herbal	5	>100	2237/2237	1085/1108	MS, RI, aroma, S
47	3-Methylthiopropanol	C_4_H_10_OS	Boiled vegetable soup	3	>10	1741/1714	1003/987	MS, RI, aroma, S
	**Nitrogen-containing compounds**							
48	2-Methylpyrazine	C_5_H_6_N_2_	Nuts, woody, characterized by soy sauce-like and savory notes	2	>10	836/827	1249/1263	MS, RI, aroma, S
49	2-Ethyl-3-methylpyrazine ^a^	C_7_H_10_N_2_	Green, roasted, nuts, with roasted and smoky characteristics	5	>100	-/-	1162/-	MS, RI, aroma, S
50	2,6-Dimethylpyrazine	C_6_H_8_N_2_	Sunflower seed, characterized by savory and vegetable-like aroma	2	>10	1330/1319	876/885	MS, RI, aroma, S
51	2-Acetyl-1-pyrroline	C_6_H_9_NO	Rice	2	>1	1358/1317	1050/1072	MS, RI, aroma, S
52	2-Ethylpyrazine ^a^	C_6_H_8_N_2_	Roasted, woody with meaty and savory characteristics	2	>10	1349/1323	954/929	MS, RI, aroma, S
53	2,3-Dimethylpyrazine ^a^	C_6_H_8_N_2_	Roasted, cocoa, featuring meaty, savory, milky, and smoky note	2	>10	1365/1335	916/919	MS, RI, aroma, S
54	2-Ethyl-6-methylpyrazine ^a^	C_7_H_10_N_2_	Roasted nuts, characterized by a roasted aroma	2	>10	1382/1375	986/1008	MS, RI, aroma, S
55	2,6-Diethylpyrazine ^a^	C_8_H_12_N_2_	Nuts	2	>10	1436/1444	1061/1090	MS, RI, aroma, S
56	2-Acetylpyridine ^a^	C_7_H_7_NO	Popcorn, roasted nut	2	>1	1621/1618	1066/1057	MS, RI, aroma, S
57	5-Methyl-2-acetylpyrazine ^a^	C_7_H_8_N_2_O	Popcorn	2	>1	1736/1723	1089/1088	MS, RI, aroma, S
58	2-Acetyl-1*H*-pyrrole	C_6_H_7_NO	Sunflower seed	2	>10	1940/1952	1045/1072	MS, RI, aroma, S
59	1H-pyrrole-2-carbaldehyde ^a^	C_5_H_5_NO	Roasted, coffee	5	>100	2036/2030	1055/1030	MS, RI, aroma
60	3-Phenylpyridine ^a^	C_11_H_9_N	Medicine	1	>10	2238/2247	1443/1470	MS, RI, aroma
61	2-Phenylethylpyrazine ^a^	C_12_H_12_N_2_	Nuts	1	>1	2381/2351	1577/1552	MS, RI, aroma, S
	**Phenolic compounds**							
62	2-Methoxyphenol	C_7_H_8_O_2_	Smoke, sweet, medicine	5	>100	1887/1860	1092/1092	MS, RI, aroma, S
63	2-Methylphenol	C_7_H_8_O	Leather	5	>100	2012/2020	1090/1068	MS, RI, aroma, S
64	4-Ethyl-2-methoxyphenol	C_9_H_12_O_2_	Clove	5	>100	2022/2033	1279/1287	MS, RI, aroma, S
65	4-Methylphenol	C_9_H_11_NO_2_	Feces	5	>100	2086/2093		MS, RI, aroma, S
66	4-Ethylphenol	C_8_H_10_O	Dry soil, animals	5	>100	2167/2167	1150/1163	MS, RI, aroma, S
67	2-Methoxy-4-vinylphenol	C_9_H_10_O_2_	Woody	5	>100	2203/2180	1330/1315	MS, RI, aroma, S
	**Furans**							
68	2-Acetyl-5-methylfuran	C_7_H_8_O_2_	Sunflower seed, woody	2	>1	1597/1605	980/977	MS, RI, aroma, S

^a^ Newly identified odorants in Huangjiu. ^b^ “cRI/lRI”, denoting the determined and reported linear retention indices, respectively. Calculated RIs were obtained on DB-WAX and DB-5 columns using n-alkanes (C5–C25), whereas literature RIs were sourced from NIST 2022. ^c^ The aroma compound was classified through comparison of its mass spectrum in library (MS), retention indices (RIs) obtained on DB-FFAP and DB-5 capillary columns, odor description (aroma), and mass spectrum with the corresponding data of authentic reference standards (S).

**Table 2:** Concentrations of 57 odor-active compounds in three Jimo Huangjius.

No.	Compounds	Concentrations (μg/L)			
		JM-G	JM-BT	JM-T	Average
1	Ethyl 2-methylpropionate ^ac^	2.27 ± 0.09	0.87 ± 0.01	1.13 ± 0.17	1.42
2	4-Methylphenol ^c^	154.24 ± 13.12	166.95 ± 11.33	327.27 ± 32.99	216.15
3	*β*-Phenylethanol ^ac^	38.67 ± 3.85	18.92 ± 1.93	30.31 ± 2.66	29,306.76
4	2-Ethyl-6-methylpyrazine ^cf^	ND	199.47 ± 15.77	869.31 ± 64.72	534.39
5	4-Ethyl-2-methoxyphenol ^c^	78.92 ± 5.02	133.01 ± 15.93	1795.67 ± 164.33	669.2
6	2-Methylpyrazine ^c^	49.57 ± 3.86	44.12 ± 4.61	91.08 ± 17.46	61.59
7	2-Methoxyphenol ^c^	22.46 ± 1.39	69.12 ± 10.02	ND	45.79
8	2-Methylphenol ^b^	3.31 ± 0.21	22.36 ± 1.80	27.25 ± 1.13	17.64
9	2-Hydroxy-3-methyl-2-cyclopentenone ^b^	41.01 ± 1.81	153.63 ± 4.10	254.38 ± 14.82	149.67
10	2,3-Dimethylpyrazine ^cf^	20.38 ± 1.94	12.57 ± 2.54	31.48 ± 3.55	21.48
11	2-Ethyl-3-methylpyrazine ^bf^	30.59 ± 2.44	13.67 ± 2.77	42.58 ± 30.11	28.95
12	3-Phenylpropionic acid ^b^	28.60 ± 1.48	110.65 ± 0.72	105.89 ± 11.57	81.71
13	4,5-Dimethyl-3-hydroxy-2,5-dihydrofuran-2-one ^b^	ND	ND	189.89 ± 3.24	189.89
14	Ethyl hexadecanoate ^c^	523.14 ± 20.93	1042.75 ± 191.32	1005.42 ± 125.39	857.1
15	3-Methylthiopropanol ^c^	10.44 ± 1.14	13.36 ± 3.69	62.88 ± 9.38	28.89
16	2-Furanmethanol	62.14 ± 6.35	271.11 ± 79.85	272.20 ± 27.52	201.82
17	3-Methylbutyric acid ^ac^	80.99 ± 7.06	18.30 ± 2.58	82.224.8 ± 9.68	60.5
18	2-Ethylpyrazine ^cf^	9.66 ± 1.21	4.09 ± 3.00	18.47 ± 14.04	10.74
19	1,1-Diethoxyethane ^ac^	21.52 ± 2.38	11.37 ± 1.06	13.89 ± 1.45	15.6
20	Ethyl 3-phenylpropionate ^c^	846.55 ± 59.39	968.29 ± 123.1	951.29 ± 186.62	922.04
21	Ethyl butyrate ^ac^	1.74 ± 0.28	1.70 ± 0.20	2.25 ± 0.24	1.9
22	2-Methoxy-4-vinylphenol ^b^	3.97 ± 0.47	9.37 ± 0.58	41.31 ± 1.83	18.22
23	Ethyl acetate ^ace^	45.44 ± 3.18	43.75 ± 5.41	27.03 ± 0.72	38.74
24	Benzaldehyde ^ace^	2.50 ± 0.29	0.84 ± 0.06	0.96 ± 0.16	1.43
25	2,6-Dimethylpyrazine ^ce^	808.22 ± 31.86	939.3 ± 42.65	1795.67 ± 164.33	1181.06
26	Ethyl phenylacetate ^ce^	200.13 ± 13.65	79.26 ± 12.26	140.57 ± 18.66	139.99
27	Butyric acid ^be^	238.50 ± 21.98	260.57 ± 2.00	790.80 ± 45.87	429.96
28	Nonanoic acid ^ce^	1593.17 ± 13.61	1754.52 ± 116.09	1787.63 ± 132.95	1711.77
29	Ethyl benzoate ^ce^	50.33 ± 3.76	59.35 ± 5.77	61.78 ± 3.44	57.15
30	Diethyl succinate ^ce^	780.44 ± 52.92	198.46 ± 14.24	308.88 ± 29.69	429.26
31	Acetic acid ^ce^	351.26 ± 36.17	389.20 ± 30.59	275.51 ± 33.00	338.66
32	2-Methylpropionic acid ^be^	200.56 ± 5.41	174.51 ± 7.22	220.06 ± 14.78	198.38
33	Pentanoic acid ^be^	25.62 ± 1.63	72.45 ± 2.94	353.19 ± 25.01	150.42
34	Hexanoic acid ^ce^	75.36 ± 6.30	122.84 ± 13.90	129.97 ± 11.72	109.39
35	Octanoic acid ^ce^	23.11 ± 1.67	30.39 ± 2.48	66.19 ± 2.93	39.9
36	Decanoic acid ^ce^	2.23 ± 0.71	14.47 ± 3.21	61.66 ± 3.74	26.12
37	Furfural ^ce^	543.18 ± 37.37	358.59 ± 25.96	505.00 ± 66.42	468.92
38	2-Phenyl-2-butenal ^ce^	279.47 ± 23.07	181.36 ± 16.20	246.35 ± 17.30	235.73
39	2-Acetyl-1*H*-pyrrole ^ce^	589.8 ± 44.23	476.43 ± 11.06	1165.87 ± 84.32	744.03
40	4-Ethylphenol ^ce^	24.39 ± 2.47	20.09 ± 1.41	61.07 ± 6.84	35.18
41	2-Methyl-1-propanol ^be^	3093.85 ± 17.41	1731.54 ± 55.69	3001.37 ± 245.09	2608.92
42	2/3-Methyl-1-butanol ^ce^	1633.05 ± 128.20	675.32 ± 44.18	889.37 ± 73.78	1065.91
43	Ethyl propionate ^ace^	1.48 ± 0.10	ND	2.79 ± 0.19	2.15
44	Cyclopentanone ^cef^	41.75 ± 3.93	53.04 ± 3.61	49.40 ± 6.08	48.06
45	3-Methyl-2-cyclopenten-1-one ^bdf^	ND	17.51 ± 0.12	8.36 ± 0.55	12.94
46	1-(Furan-2-yl) propan-1-one ^cd^	74.74 ± 25.70	ND	517.35 ± 17.36	296.05
47	1-(2-Furyl)-ethanone ^cef^	385.72 ± 1.27	400.84 ± 33.94	1147.1 ± 95.10	644.55
48	5-Ethoxy-4,5-dihydro-2(3*H*)-furanone ^cd^	46.19 ± 1.76	ND	37.79 ± 4.25	41.99
49	2-Hydroxycyclopent-2-en-1-one ^cdf^	ND	2.70 ± 0.27	6.18 ± 0.38	4.44
50	4-Phenyl-2-butanone ^cef^	ND	ND	9.58 ± 1.37	9.58
51	3-Ethyl-2-hydroxycyclopent-2-en-1-one ^cd^	9.65 ± 1.08	60.57 ± 7.79	91.53 ± 5.99	53.92
52	1-(2-Furyl)-2-hydroxyethanone ^cd^	ND	ND	10.58 ± 1.55	10.58
53	1,1-Diethoxy-3-methylbutane ^cef^	283.88 ± 28.51	134.84 ± 15.51	325.08 ± 35.44	247.93
54	Furan-2,5-dicarbaldehyde ^bef^	6.84 ± 0.59	13.35 ± 0.53	1.34 ± 0.14	7.18
55	2,6-Diethylpyrazine ^bef^	ND	ND	0.61 ± 0.01	0.61
56	1*H*-Pyrrole-2-carbaldehyde ^bdf^	1.38 ± 0.32	4.21 ± 0.04	6.67 ± 0.74	4.09
57	3-Phenylpyridine ^bdf^	2.80 ± 0.05	0.94 ± 0.09	8.04 ± 0.51	3.93

^a^ The concentration was mg/L. ^b^ Quantified by gas chromatography–mass spectrometry combined with solid-phase extraction (SPE-GC/MS). ^c^ Quantified by gas chromatography–mass spectrometry combined with headspace solid-phase microextraction (HS-SPME-GC/MS). ^d^ These aroma-active compounds were semi-quantified by means of an internal standard method. ^e^ These compounds were quantified via standard curves. The standard curves, as well as the qualitative and quantitative ions, are provided in Appendix A (Table A2). ^f^ These aroma-active compounds were newly quantified in Huangjiu.

**Table 3:** Odor thresholds and odor activity values of key odorants in Jimo Huangjiu.

	Odoants	Thresholds (μg/L) ^a^	OAVs ^b^			
			JM-G	JM-BT	JM-T	Average
1	Ethyl 2-methylpropionate	0.000015	151,182,667	58,073,333	75,626,000	94,960,667
2	4-Methylphenol	0.000008	19,280,000	20,868,750	40,908,750	27,019,167
3	*β*-Phenylethanol	0.002	19,336,760	9,464,170	15,159,210	14,653,380
4	2-Ethyl-6-methylpyrazine	0.00004	-	4,986,750	21,732,750	8,906,500
5	4-Ethyl-2-methoxyphenol	0.0002	394,600	665,050	8,978,350	3,346,000
6	2-Methylpyrazine	0.00006	826,167	735,333	1,518,000	1,026,500
7	2-Methoxyphenol	0.00003	748,667	2,304,000	-	1,017,556
8	2-Methylphenol	0.000031	106,774	721,290	879,032	569,032
9	2-Hydroxy-3-methyl-2-cyclopentenone	0.0003	136,700	512,100	847,933	498,911
10	2,3-Dimethylpyrazine	0.00008	254,750	157,125	393,500	268,458
11	2-Ethyl-3-methylpyrazine	0.00013	235,308	105,154	327,538	222,667
12	3-Phenylpropionic acid	0.0005	57,200	221,300	211,780	163,427
13	4,5-Dimethyl-3-hydroxy-2,5-dihydrofuran-2-one	0.0005	-	-	379,780	126,593
14	Ethyl hexadecanoate	0.014	37,367	74,482	71,816	61,222
15	Methionol	0.0015	6960	8907	41,920	19,262
16	2-Furanmethanol	0.015	4143	18,074	18,147	13,454
17	3-Methylbutyric acid	8	10,122	2288	10,278	7563
18	2-Ethylpyrazine	0.004	2415	1023	4618	2685
19	1,1-Diethoxyethane	50	431	227	278	312
20	Ethyl 3-phenylpropionate	5	169	194	190	184
21	Ethyl butyrate	20	87	85	113	95
22	2-Methoxy-4-vinylphenol	40	7	10	10	9
23	Ethyl acetate	15,000	3	3	2	3
24	Benzaldehyde	515	5	2	2	3
25	2,6-Dimethylpyrazine	400	2	2	4	3
26	Ethyl phenylacetate	73	3	1	2	2
27	Butyric acid	173	1	2	5	2
28	Nonanoic acid	1100	1	2	2	2
29	Ethyl benzoate	575	<1	<1	<1	<1
30	Diethyl succinate	14,417.5	<1	<1	<1	<1
31	Acetic acid	24,000	<1	<1	<1	<1
32	2-Methylpropionic acid	2300	<1	<1	<1	<1
33	Pentanoic acid	11,000	<1	<1	<1	<1
34	Hexanoic acid	806.5	<1	<1	<1	<1
35	Octanoic acid	500	<1	<1	<1	<1
36	Decanoic acid	15,000	<1	<1	<1	<1
37	Furfural	14,100	<1	<1	<1	<1
38	2-Phenyl-2-butenal	20,000	<1	<1	<1	<1
39	2-Acetyl-1*H*-pyrrole	58,600	<1	<1	<1	<1
40	4-Ethylphenol	140	<1	<1	<1	<1
41	2-Methyl-1-propanol	40,000	<1	<1	<1	<1
42	2/3-Methyl-1-butanol	30,000	<1	<1	<1	<1
43	Ethyl propionate	1800	<1	<1	<1	<1
44	Cyclopentanone	51,100	<1	<1	<1	<1
45	3-Methyl-2-cyclopenten-1-one	-	-	-	-	-
46	1-(Furan-2-yl) propan-1-one	-	-	-	-	-
47	1-(2-Furyl)-ethanone	10,000	<1	<1	<1	<1
48	5-Ethoxy-4,5-dihydro-2(3*H*)-furanone	-	-	-	-	-
49	2-Hydroxycyclopent-2-en-1-one	-	-	-	-	-
50	4-Phenyl-2-butanone	2500	<1	<1	<1	<1
51	3-Ethyl-2-hydroxycyclopent-2-en-1-one	-	-	-	-	-
52	1-(2-Furyl)-2-hydroxyethanone	-	-	-	-	-
53	1,1-Diethoxy-3-methylbutane	3000	<1	<1	<1	<1
54	Furan-2,5-dicarbaldehyde	5000	<1	<1	<1	<1
55	2,6-Diethylpyrazine	6	<1	<1	<1	<1
56	1*H*-Pyrrole-2-carbaldehyde	-	-	-	-	-
57	3-Phenylpyridine	-	-	-	-	-

^a^ Odor thresholds from reference [42]. When multiple threshold values were available, we prioritized those determined in matrices more comparable to Huangjiu, such as beer, wine, or aqueous ethanol solutions (5–15%, *v*/*v*). -, indicated as the threshold value at which the substance was not detected in literatures. ^b^ OAVs represents the odorant activity value; JM-G, JM-BT, and GM-T, respectively, represent dry, semi-sweet, and sweet type Huangjius.

**Table 4:** Addition experiments results.

No.	Group		Alcohol	Daqu	Woody	Aged	Sweet	Acidic	Smoky	Burnt-like
1	Control group 1	Jimo sweet Huangjiu original sample	3.82	3.91	2.73	2.45	2.91	3.27	5.82	7.73
2	Addition group 1	2-methoxyphenol	-	-	-	-	4.17 **	-	8.33 ***	10.80 ***
3	Control group 2	Jimo dry Huangjiu original sample	3.00	3.50	3.50	5.50	4.00	4.00	4.50	4.5
4	Addition group 2_1	4-methylphenol	-	-	-	-	6.7 **	-	5.4 ***	6.33 ***
5	Addition group 2_2	2-methylphenol	-	-	-	-	-	-	5.875 ***	6.17 ***
6	Addition group 2_3	4-ethyl-2-methoxyphenol	-	-	-	-	-	-	7.91 ***	-
7	Addition group 2_4	2-methoxy-4-vinylphenol	-	-	-	-	-	-	6.00 ***	6.50 ***
8	Addition group 2_5	2-methylpyrazine	-	-	-	-	6.33 ***	-	-	6.00 ***
9	Addition group 2_6	2-ethylpyrazine	-	-	5.63	-	-	-	6.33 ***	-
10	Addition group 2_7	2,3-dimethylpyrazine	-	-	-	-	-	-	6.10 ***	5.88 ***
11	Addition group 2_8	2,6-dimethylpyrazine	-	-	-	-	-	-	6.50 ***	-
12	Addition group 2_9	2-ethyl-3-methylpyrazine	-	-	-	-	6	-	6.37 ***	-
13	Addition group 2_10	2-ethyl-6-methylpyrazine	-	-	6.50 ***	-	5.25 **	-	-	-
14	Control group 3	Jimo dry Huangjiu original sample	3.00	3.50	3.50	5.50	4.00	4.00	4.50	4.50
15	Addition group 3_1	4-ethylphenol	-	-	-	7.50 ***		5.87 ***	5.83 ***	-
16	Addition group 3_2	4-ethylphenol + nonanoic acid	-	5.50 **	-	-	-	7.00 ***	6.17 ***	8.00 ***
17	Addition group 3_3	nonanoic acid	-	5.33 **	6.00 ***	-	-	6.83 ***	6.33 ***	-

**, highly significant (*p* ≤ 0.01); ***, very highly significant (*p* ≤ 0.001).

## Data Availability

The original contributions presented in this study are included in the article. Further inquiries can be directed to the corresponding authors.

## Abbreviations

The following abbreviations are used in this manuscript:

CATA	Check-All-That-Apply
fast-track AEDA	Fast-track Aroma extract dilution analysis
GC-O-MS	Gas chromatography-olfactometry-mass spectrometry
JM-BT	Jimo semi-sweet type
JM-G	Jimo dry type Huangjiu
JMHJ	Jimo Huangjiu
JM-T	Jimo sweet type Huangjiu
Osme	Odor-specific magnitude estimation
QDA	Quantitative Descriptive Analysis
NCCs	Nitrogen-containing compounds

## Appendix A

**Chemicals.** The majority of compounds were procured from J&K Scientific Ltd. (Beijing, China), including: 1-(2-furyl)-1-propanone (96%), 1-(2-furyl)-2-hydroxyethanone (95%), 1-(2-methylcyclopenten-1-yl)ethanone (98%), 1,1-diethoxy-3-methylbutane (>95%), 1*H*-pyrrole-2-carbaldehyde (97%), 1-octen-3-one (97%), 2/3-methyl-1-butanol (99%), 2,3-dimethylpyrazine (98%), furan-2,5-dicarbaldehyde (99.5%), 2,6-diethylpyrazine (95%), 2,6-dimethylpyrazine (98%), 2-acetyl-1-pyrroline (>98%), 2-acetyl-5-methylfuran (98%), 2-acetylpyridine (98%), 2-ethyl-3-methylpyrazine (99%), 2-ethyl-6-methylpyrazine (95%), 2-ethylpyrazine (97%), 2-hydroxy-3-methyl-2-cyclopentenone (98%), 2-hydroxycyclopent-2-en-1-one (97%), 2-methoxy-4-vinylphenol (97%), 2-methoxyphenol (98%), 2-methyl-1-propanol (99%), 2-methylpyrazine (98%), 2-phenyl-2-butenal (97%), 3-methyl-2-cyclopenten-1-one (98%), 3-phenylpropionic acid (99%), 3-phenylpyridine (97%), 4-ethyl-2-methoxyphenol (98%), 4-ethylphenol (98%), 4-methylphenol (97.6%), 4-phenyl-2-butanone (99%), acetic acid (99.5%), benzaldehyde (98%), butyric acid (99%), cyclopentanone (99%), decanoic acid (98%), diethyl malate (97%), diethyl succinate (99%), ethyl acetate (99.5%), ethyl benzoate (99%), ethyl butyrate (99%), ethyl phenylacetate (99%), ethyl phenylpropanoate (97%), ethyl propionate (99%), furfural (99%), hexanoic acid (98%), nonanoic acid (98%), octanoic acid (98%), pentanoic acid (98%), *β*-phenethyl alcohol (99%), and 1,1-diethoxyethane (99.5%).

Other reagents were obtained from the following commercial suppliers: 1-(1*H*-Pyrrole-2-yl)-ethanone (99%) from Wuhan Haorong Biotechnology Co., Ltd. (Wuhan, China); 1-(2-Furyl)-ethanone (99%) from Wuhan Jushun Chemical Co., Ltd. (Wuhan, China); 2-Furanmethanol (≥99.5%) from Hubei Tuoyuan Fine Chemical Co., Ltd. (Wuhan, China); 2-Methylphenol (99.5%) from Shandong Senrong Chemical Co., Ltd. (Zibo, China); 2-Phenylethylpyrazine (98%) from Zhengzhou Alpha Chemical Co., Ltd. (Zhengzhou, China); 3-Hydroxybutanone (98%) from Hubei Maoerwo Biomedical Co., Ltd. (Wuhan, China); 3-Methylthiopropanol (99%) from Jingzhou Yinjie Chemical Co., Ltd. (Jingzhou, China); 4,5-Dimethyl-3-hydroxy-2,5-dihydrofuran-2-one (99%) from Hubei Chenghai Chemical Co., Ltd. (Tianmen, China); 5-Ethoxydihydro-2(3H)-furanone (98%) from Shanghai Bide Pharmaceutical Technology Co., Ltd. (Shanghai, China); 5-Methyl-2-acetylpyrazine (98%) from TCI (Shanghai) Development Co., Ltd. (Shanghai, China); Ethyl 2-hydroxy-3-methylbutyrate (98%) from Shanghai Macklin Biochemical Co., Ltd. (Shanghai, China); Ethyl 2-methylpropionate (99%) from Jiangsu Leien Environmental Protection Technology Co., Ltd. (Nantong, China); Ethyl 3-methylbutyrate (99%) from Hubei Xinjing New Materials Co., Ltd. (Wuhan, China); and Ethyl hexadecanoate (99%) from Henan Wokas Biotechnology Co., Ltd. (Xinyang, China).

**Table A1:** Sensory evaluation criteria of quantitative descriptive analysis.

Descriptor	Reference Standard	Score ^b^	Reference Concentration
Smoky	2,6-Dimethylphenol	9.0	1000 µg/L
		6.0	300 µg/L
		3.0	100 µg/L
Sweet	Ethyl 3-phenylpropionate ^a^	9.0	1200 μg/L
		6.0	600 μg/L
		3.0	300 μg/L
Chen-aroma	Aged vinegar ^a^	9.0	100 mL/L
		6.0	50 mL/L
		3.0	25 mL/L
Alcoholic	Ethanol aqueous solution	9.0	30% ethanol
		6.0	20% ethanol
		3.0	10% ethanol
Acidic	Acetic acid ^a^	9.0	500 mg/L
		6.0	50 mg/L
		3.0	5 mg/L
Qu-aroma	Daqu (fermentation starter) ^a^	9.0	300 g/L
		6.0	150 g/L
		3.0	75 g/L
Woody	Guaiacol	9.0	1000 µg/L
		6.0	100 µg/L
		3.0	10 µg/L
Burnt-like	Methylcyclopentenolone	9.0	1000 µg/L
		6.0	100 µg/L
		3.0	10 µg/L

^a^ These reference standards were dissolved in a 15% (*v*/*v*) aqueous ethanol solution. ^b^ A score of 3.0 indicates a weak intensity, 6.0 indicates a moderate intensity, and 9.0 indicates an extremely strong intensity.

**Table A2:** Standard curves of 57 odor-active compounds in three Jimo Huangjius.

No.	Compounds	R^2^	Standard Curves	Quatify Ion	Quantify Ion
1	Ethyl 2-methylpropionate	0.9996	y = 17.087x + 0.2034	43, 71, 116	43
2	4-Methylphenol	0.9995	y = 4.3767x − 0.4947	77, 107, 108	107
3	*β*-Phenylethanol	0.9960	y = 26.449x − 0.8361	65, 91, 92, 122	91
4	2-Ethyl-6-methylpyrazine	0.9991	y = 9.4792x + 0.0884	94,121,122	121
5	4-Ethyl-2-methoxyphenol	0.9980	y = 3.7617x + 0.0534	122, 137, 152	137
6	2-Methylpyrazine	0.9998	y = 4.5675x + 0.0083	53, 67, 94	94
7	2-Methoxyphenol	0.9978	y = 4.3361x − 0.7808	81,109,124	109
8	2-Methylphenol	0.9992	y = 2.1272x − 0.2227	79,107,108	108
9	2-Hydroxy-3-methyl-2-cyclopentenone	0.9943	y = 802.11x − 0.1499	55, 69, 112	112
10	2,3-Dimethylpyrazine	0.9999	y = 10.245x + 0.0047	42, 67,108	67
11	2-Ethyl-3-methylpyrazine	0.9999	y = 6.3336x + 0.0254	67, 121, 122	121
12	3-Phenylpropionic acid	0.9997	y = 3.1898x − 0.1101	91, 104, 150	91
13	4,5-Dimethyl-3-hydroxy-2,5-dihydrofuran-2-one	0.9975	y = 4.9874x + 5.4398	55, 83, 128	83
14	Ethyl hexadecanoate	0.9993	y = 10.55x + 0.0499	43, 88, 101	88
15	3-Methylthiopropanol	0.9992	y = 4.5408x − 0.4899	58, 61, 106	106
16	2-Furanmethanol	0.9983	y = 3.6956x + 0.1723	41, 53, 98	98
17	3-Methylbutyric acid	0.9985	y = 832.00x + 0.7641	41, 43, 60	60
18	2-Ethylpyrazine	0.9999	y = 2.0986x + 0.8763	80, 107, 108	107
19	1,1-Diethoxyethane	0.9935	y = 15.352x − 0.3681	45, 47, 73	45
20	Ethyl 3-phenylpropionate	0.9996	y = 4.3406x − 0.2912	91, 104, 107	104
21	Ethyl butyrate	0.9978	y = 8.6171x + 0.2336	43, 71, 88	71
22	2-Methoxy-4-vinylphenol	0.9955	y = 1468.2x − 0.0961	77, 107, 135, 150	135
23	Ethyl acetate	0.9969	y = 60.371x + 0.282	43, 45, 61	43
24	Benzaldehyde	0.9982	y = 3.1438x − 0.0648	77, 105, 106	77
25	2,6-Dimethylpyrazin	0.9998	y = 26.596x + 0.0634	41,42,108, 109	108
26	Ethyl phenylacetate	0.9999	y = 4.2555x + 0.2873	65, 91, 92, 164	91
27	Butyric acid	0.9982	y = 6.6183x − 0.5812	41, 60, 73	60
28	Nonanoic acid	0.9932	y = 21.345x + 1.477	57, 60, 73	60
29	Ethyl benzoate	0.9927	y = 0.3953x + 0.0222	77, 105, 122	105
30	Diethyl succinate	0.998	y = 2.3028x − 0.7764	55, 101, 129	101
31	Acetic acid	0.9989	y = 10.302x − 0.2952	43, 45, 60	43
32	2-Methylpropionic acid	0.9985	y = 6.8109x − 0.541	41, 43, 73	43
33	Pentanoic acid	0.9929	y = 7.3948x − 1.0927	41, 60, 73	60
34	Hexanoic acid	0.9928	y = 5.5422x − 1.0946	60, 73, 87	60
35	Octanoic acid	0.9981	y = 7.4343x − 1.3475	43, 60, 73	60
36	Decanoic acid	0.9995	y = 2.9806x − 0.5337	41, 60, 73	60
37	Furfural	0.997	y = 5.6498x − 0.7408	67, 95, 96	96
38	2-Phenyl-2-butenal	0.9988	y = 2.5724x + 0.1181	115, 117, 146	117
39	2-Acetyl-1*H*-pyrrole	0.9938	y = 22.459x + 0.1127	66, 94, 109	94
40	4-Ethylphenol	0.9987	y = 2.3857x − 0.2444	77, 107, 122	107
41	2-Methyl-1-propanol	0.992	y = 11.99x − 1.1309	41, 42, 43	43
42	2/3-Methyl-1-butanol	0.9931	y = 3.1157x − 1.1488	55, 70,42	55
43	Ethyl propionate	0.9991	y = 37.904x + 0.1545	57, 75, 102	57
44	Cyclopentanone	0.9993	y = 13.545x + 0.2456	41, 55, 84	55
45	3-Methyl-2-cyclopenten-1-one	0.9993	y = 18.423x + 1.3345	53, 67, 96	96
46	1-(Furan-2-yl) propan-1-one	0.9994	y = 16.342x + 0.3356	95, 96, 124	95
47	1-(2-Furyl)-ethanone	0.9980	y = 24.474x + 0.1243	43, 95,110	95
48	5-Ethoxy-4,5-dihydro-2(3*H*)-furanone	0.9998	y = 15.333x + 0.2397	85, 58,56	85
49	2-Hydroxycyclopent-2-en-1-one	0.9980	y = 19.445x + 0.2378	42, 55, 98	98
50	4-Phenyl-2-butanone	0.9980	y = 8.8654x + 0.7854	43,105, 148	43
51	3-Ethyl-2-hydroxycyclopent-2-en-1-one	0.9980	y = 16.448x + 1.4478	55, 83, 126	126
52	1-(2-Furyl)-2-hydroxyethanone	0.9980	y = 20.447x + 0.8975	95, 96, 126	95
53	1,1-Diethoxy-3-methylbutane	0.9999	y = 0.6889x + 0.0219	47, 71, 103	103
54	Furan-2,5-dicarbaldehyde	0.9980	y = 4.6673x + 0.5653	124, 123, 95	124
55	2,6-Diethylpyrazine	0.9980	y = 6.8854x + 0.9267	108, 135, 136	135
56	1*H*-Pyrrole-2-carbaldehyde	0.9997	y = 95.737x + 0.1109	66, 94, 95	95
57	3-Phenylpyridine	0.9997	y = 39.454x + 1.6576	154, 155, 156	155
